# Polycythemia vera as a presentation of renal angiomyolipoma: a case report

**DOI:** 10.1186/1752-1947-3-90

**Published:** 2009-10-31

**Authors:** Ming-Shyan Lin, Yu-Shin Hung, Hsueh-Hua Wu, Ming-Chung Kuo, Tzu-Fang Shiu, Cheng-Keng Chuang, Lee-Yung Shih, Pao-Hsien Chu

**Affiliations:** 1 Division of Cardiology, Department of Internal Medicine, Chang Gung Memorial Hospital, Chang Gung University College of Medicine, Taipei, Taiwan; 2 Division of Hematology, Chang Gung Memorial Hospital, Chang Gung University College of Medicine, Taipei, Taiwan; 3 Division of Urology, Chang Gung Memorial Hospital, Chang Gung University College of Medicine, Taipei, Taiwan

## Abstract

**Introduction:**

Angiomyolipoma is a common benign renal tumor composed of thick-walled blood vessels, smooth muscle, and adipose tissue. It may be found incidentally during workup for suspected renal disease. Although angiomyolipoma may present as a palpable, tender renal mass with flank pain and gross or microscopic hematuria, many patients are asymptomatic. Erythrocytosis is an unusual presentation, and malignant transformation may be suspected. This report describes a rare case of a woman diagnosed with renal angiomyolipoma and polycythemia vera. The report discusses the differential diagnosis using erythropoietin, erythropoietin-receptor and Janus kinase 2.

**Case presentation:**

A 79-year-old Chinese woman was diagnosed with erythrocytosis according to World Health Organization criteria. An upper left renal pole angiomyolipoma was successfully ablated after multiple phlebotomy treatments. Red cell count immediately returned to normal, but gradually increased after 4 months. Polycythemia vera was finally diagnosed by positive mutation of Janus kinase 2 and negative erythropoietin protein expression. Her clinical symptoms improved with regular phlebotomy and hydroxyurea treatment.

**Conclusion:**

Concurrent occurence of angiomyolipoma and polycythemia vera is rare. Polycythemia vera can be easily missed. Polycythemia vera can be confirmed with high specificity and sensitivity by the acquired somatic mutation. Surgical intervention for this renal tumor should be avoided unless malignancy or renal cell carcinoma is suspected or to prevent spontaneous rupture of larger tumors.

## Introduction

Polycythemia vera is a rare presentation of renal angiomyolipoma [1]. Angiomyolipoma is a mixed mesenchymal tumor belonging to the family of perivascular epithelioid cell tumors (PEComa). Renal angiomyolipoma can be found incidentally during workup for suspected renal disease and is one of the few lesions which can be specifically diagnosed by radiological findings. Although angiomyolipoma may present as a palpable, tender renal mass with flank pain and gross or microscopic hematuria, many patients are asymptomatic. Moreover, the recent discovery of Janus kinase 2 (JAK2) V617F mutation in most patients with polycythemia vera opens new avenues for the treatment of this disease.

This report describes a rare case of a woman diagnosed with renal angiomyolipoma and polycythemia vera and its differential diagnosis by erythropoietin (EPO), EPO-receptor (EPO-R) [1] and JAK2.

## Case presentation

This is a case report of a 79-year-old Chinese woman with a 5-year history of hypertension but no history of body weight loss, systemic diseases or smoking. She had suffered from palmer erythema and facial flushing associated with itching for several years, and had undergone phlebotomy for erythrocytosis several times before visiting our clinic. However, due to persistent leukocytosis (169,000/μL, normal <10,000/μL), erythrocytosis (red blood cells 7.00 million/μL, hemoglobin 18.3 g/dL, hemocrit 57.7%) and thrombocytosis (915,000/μL), she was transferred to the hematology department for evaluation of possible polycythemia vera.

Red cell volume measured by Cr-51 tagged cells was 2698.2 mL (equivalent to 51.6 mL/kg; normal range: 20-30 mL/kg). The calculated total and blood plasma volumes were 5195.8 mL (equivalent to 99.5 mL/kg; normal range: 50-75 mL/kg) and 2497.6 mL (equivalent to 47.8 mL/kg; normal range: 30-45 mL/kg), respectively. Erythrocytosis was diagnosed according to criteria for absolute erythrocytosis (female with blood plasma volume of ≥32 mL/kg and leukocyte alkaline phosphatase score of 167), and hydroxyurea (500 mg per day) was started. Urine vanillylmandelic acid was 7.5 mg/day (within the normal range).

A renal echogram revealed bilateral normal-sized kidneys as well as an oval hyperechogenic tumor (3.9 × 2.0 cm) without acoustic shadow in the left adrenal area. Whole body computed tomography revealed a left renal tumor without lymphadenopathy. Left partial nephrectomy was performed 3 months later after satisfactory control of blood pressure. The pathology specimen was a kidney segment measuring 4.5 × 3.7 × 1.7 cm and weighing 15.2 g. The specimen consisted almost entirely of soft, yellow tumor. The tumor contained mature adipose tissue, thick-walled vessels and fascicles of smooth muscle cells. The resection margins were tumor-free.

**Figure 1:**
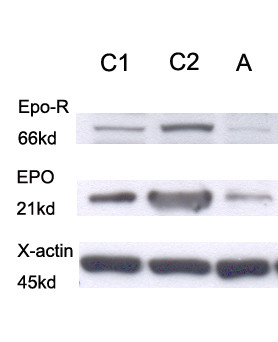
**Western blot analysis showing reduced EPO and EPO-R expression in this patient with angiomyolipoma and polycythemia vera (C1 and C2 are normal control kidney tissues, and A is angiomyolipoma tissue)**.

After surgery, her hemoglobin returned to normal for 4 months and then recurrent polycythemia vera was again treated by regular phlebotomy and hydroxyurea treatment. Western blot analysis showed relatively decreased EPO and EPO-R levels (Figure 1) in the angiomyolipoma. Moreover, the positive JAK2 (V617F) point mutation was demonstrated using the DNA from granulocytes in the peripheral blood.

Primers for JAK2 (V617F) point mutation were designed using an ARMS design program20 and included mismatches to maximize discrimination of the 2 alleles (shown in lowercase) and mutant/wild-type-specific bases. Polymerase chain reaction (PCR) primers were: forward outer (FO), 5-TCCTCAGAACGTTGATGGCAG-3; reverse outer (RO), 5-ATTGCTTTCCTTTTTCACAAGAT-3; forward wild-type-specific (Fwt), 5-GCATTTGGTTTTAAATTATGGAGTATaTG-3; reverse-mutant-specific (Rmt), 5-GTTTTACTTACTCTCGTCTCCACAaAA-3.

## Discussion

Polycythemia vera is a chronic myeloproliferative disorder characterized (according to World Health Organization (WHO) criteria) by clonal proliferation of myeloid cells with variable morphologic maturity and hematopoietic efficiency. Elevated red blood cell mass (RCM) distinguishes polycythemia vera from other myeloproliferative disorders; but is insufficient for diagnosis. This report describes a final diagnosis by EPO expression and JAK2 mutation [2] after clinical follow-up.

Erythrocytosis may be a secondary cause in cases associated with increased RCM or EPO-secretion. EPO is secreted in chronic hypoxia and EPO-secreting tumors [3] including renal cell carcinoma, hepatocellular carcinoma, hemangioblastoma, pheochromocytoma and uterine myoma. Elevated EPO is also caused by hypoxemia in some cases of chronic pulmonary disease, shunt infection and sleep apnea. Angiomyolipoma is a benign tumor, but it also can demonstrate aggressive behavior and possible malignant transformation to sarcoma, adenocarcinoma, or renal cell carcinoma [4]. According to a previous study, larger angiomyolipomas can be easily diagnosed by ultrasonography [5] or computed tomography, and highly dense fat content within the tumor helps to make a correct diagnosis as angiomyolipoma [6]. No angiography or further biopsy would be needed. But some limitations of different diagnosis have been observed with low fat content, or in combination with hemorrhage or necrosis.

Epithelioid angiomyolipomas are distinguished from renal cell carcinomas by positive immunostaining for melanoma markers and smooth muscle cell markers. In addition, angiomyolipomas are also more commonly associated with renal cell carcinoma in tuberous sclerosis. The simultaneous presence of angiomyolipoma and renal cell carcinoma has been reported [7] and in that case it was a source of exogenous EPO secretion which was able to be corrected [8].

Acquired or inherited mutations leading to an abnormality within the erythroid progenitors identified in polycythemia vera and its rare familial variants include EPO-R mutations, which are associated with Chuvash polycythemia. Erythrocytosis has not been reported in angiomyolipoma [6]. Indeed, the level of EPO or EPO-R in the reported case was not elevated, which helped exclude secondary causes of erythrocytosis.

## Conclusion

Recent studies have shown that polycythemia vera can be confirmed by the acquired somatic mutation JAK2 (V617F). This mutation is present in almost all patients with polycythemia vera, most patients with essential thrombocythemia and idiopathic myelofibrosis and in some patients with atypical myeloproliferative disorder. As in our patient, a positive JAK2 (V617F) point mutation can be proven as 'true' polycythemia vera with high specificity and sensitivity. Surgical intervention for angiomyolipomas should be avoided unless malignancy or renal cell carcinoma is suspected or to prevent spontaneous rupture of larger tumors. The JAK2 (V617F) point mutation test is sufficient for confirming the differential diagnosis of true polycythemia vera before surgery, and surgical intervention for angiomyolipoma should be evaluated cautiously [9]. In this patient, we also provided EPO and EPO-R evaluation, which showed a reduction in levels that proved that the angiomyolipoma was not a direct source of erythrocytosis. The JAK2 (V617F) point mutation test before surgery is very important in this situation, and we would have performed elective biopsy for detection of EPO and EPO-R levels if the mutation test was negative. If EPO and EPO-R levels are negative, the patient would need to be worked-up for other secondary causes of polycythemia and consideration given to continued medical treatment for polycythemia vera after surgery.

## Abbreviations

EPO: erythropoietin; EPO-R: EPO-receptor; JAK2: Janus kinase 2; PEComa: perivascular epithelioid cell tumors; PCR: polymerase chain reaction; RCM: red blood cell mass.

## Consent

Written informed consent was obtained from the patient for publication of this case report and any accompanying images. A copy of the written consent is available for review by the Editor-in-Chief of this journal.

## Competing interests

The authors declare that they have no competing interests.

## Authors' contributions

MSL analyzed and interpreted the patient data and wrote the manuscript. YSH, MCK and LYS interpreted the patient data regarding the hematological disease. HHW and TFS analyzed and interpreted the molecular biology data. CKC interpreted the patient data regarding the kidney disease. PHC coordinated and proved the hypothesis. All authors read and approved the final manuscript.
